# Phylogeography of the pallid kangaroo mouse, *Microdipodops pallidus*: a sand-obligate endemic of the Great Basin, western North America

**DOI:** 10.1111/j.1365-2699.2008.01942.x

**Published:** 2008-11

**Authors:** John C Hafner, Nathan S Upham, Emily Reddington, Candice W Torres

**Affiliations:** 1 Moore Laboratory of Zoology and Department of Biology, Occidental CollegeLos Angeles, CA; 2 3 Cherry Street, Mansfield, MA; 3 Department of Environmental Sciences, Policy, and Management, University of CaliforniaBerkeley, CA, USA

**Keywords:** Conservation biogeography, cryptic species, directional analysis, evolutionarily significant units, Great Basin, historical biogeography, *Microdipodops pallidus*, mitochondrial DNA, pallid kangaroo mouse, phylogeography

## Abstract

**Aim:**

Kangaroo mice, genus *Microdipodops* Merriam, are endemic to the Great Basin and include two species: *M. pallidus* Merriam and *M. megacephalus* Merriam. The pallid kangaroo mouse, *M. pallidus*, is a sand-obligate desert rodent. Our principal intent is to identify its current geographical distribution and to formulate a phylogeographical hypothesis for this taxon. In addition, we test for orientation patterns in haplotype sharing for evidence of past episodes of movement and gene flow.

**Location:**

The Great Basin Desert region of western North America, especially the sandy habitats of the Lahontan Trough and those in south-central Nevada.

**Methods:**

Mitochondrial DNA sequence data from portions of three genes (16S ribosomal RNA, cytochrome *b*, and transfer RNA for glutamic acid) were obtained from 98 individuals of *M. pallidus* representing 27 general localities sampled throughout its geographical range. Molecular sequence data were analysed using neighbour-joining, maximum-parsimony, maximum-likelihood and Bayesian methods of phylogenetic inference. Directional analysis of phylogeographical patterns, a novel method, was used to examine angular measurements of haplotype sharing between pairs of localities to detect and quantify historical events pertaining to movement patterns and gene flow.

**Results:**

Collecting activities showed that *M. pallidus* is a rather rare rodent (mean trapping success was 2.88%), and its distribution has changed little from that determined three-quarters of a century ago. Two principal phylogroups, distributed as eastern and western moieties, are evident from the phylogenetic analyses (mean sequence divergence for cytochrome *b* is *c*. 8%). The western clade shows little phylogenetic structure and seems to represent a large polytomy. In the eastern clade, however, three subgroups are recognized. Nine of the 42 unique composite haplotypes are present at two or more localities and are used for the orientation analyses. Axial data from haplotype sharing between pairwise localities show significant, non-random angular patterns: a north-west to south-east orientation in the western clade, and a north-east to south-west directional pattern in the eastern clade.

**Main conclusions:**

The geographical range of *M. pallidus* seems to be remarkably stable in historical times and does not show a northward (or elevationally upward) movement trend, as has been reported for some other kinds of organism in response to global climate change. The eastern and western clades are likely to represent morphologically cryptic species. Estimated times of divergence of the principal clades of *M. pallidus* (4.38 Ma) and between *M. pallidus* and *M. megacephalus* (8.1 Ma; data from a related study) indicate that kangaroo mice diverged much earlier than thought previously. The phylogeographical patterns described here may serve as a model for other sand-obligate members of the Great Basin Desert biota.

## Introduction

Kangaroo mice, genus *Microdipodops* Merriam, belong to the rodent family Heteromyidae Gray and are restricted in distribution to sandy habitats in the Great Basin Desert of western North America. Relative to other heteromyid genera [*Perognathus* Wied-Neuwied and *Chaetodipus* Merriam (pocket mice), *Dipodomys* Gray (kangaroo rats) and *Heteromys* Desmarest (spiny pocket mice)], *Microdipodops* has an unusually small geographical distribution and is depauperate in number of species (Schmidly *et al.*, 1993; Patton, 2005; Hafner *et al.*, 2007). Only two species are currently recognized in the genus: *M. megacephalus* Merriam, the dark kangaroo mouse, and *M. pallidus* Merriam, the pallid kangaroo mouse. Kangaroo mice are also considered to be rather uncommon members of the nocturnal desert rodent community (Hall, 1941; Hafner, 1981; Hafner *et al.*, 1996).

Morphologically and ecologically, *M. pallidus* appears to be more specialized than *M. megacephalus*. Relative to its congener, *M. pallidus* has more highly inflated auditory bullae, larger hind feet, a smaller geographical distribution, and is more stenotopic (Hall, 1941; Hafner, 1981; Hafner *et al.*, 1996). Although *M. megacephalus* tolerates a variety of sandy substrates and floral associations throughout the Great Basin, *M. pallidus* is restricted typically to fine, loose, sandy soils (with little or no gravel overlay) in the lower portion of the Upper Sonoran Life Zone [usually at elevations below the sagebrush (*Artemisia* Linnaeus) community]. Hence, the pallid kangaroo mouse is a highly specialized, sand-obligate organism, and an understanding of its phylogeographical patterns may provide a model for future studies of other sand-obligate organisms in the Great Basin. We used DNA sequencing data from portions of three mitochondrial genes, 16S ribosomal RNA (16S), cytochrome *b* (*Cytb*), and transfer RNA for glutamic acid (tRNA^Glu^), to reconstruct phylogenetic relationships within *M. pallidus* and interpret those patterns in the context of historical biogeography.

## Materials and methods

### Study area, specimens examined and field work

**Figure 1:**
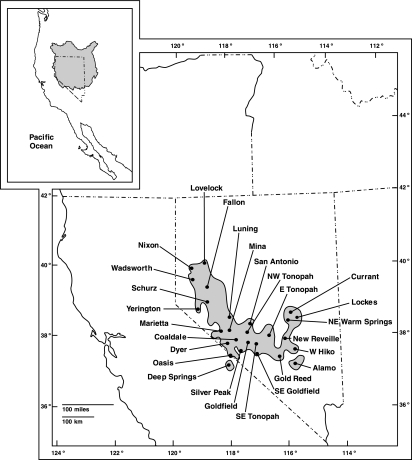
Map showing the distribution of the pallid kangaroo mouse *Microdipodops pallidus* Merriam, and the 27 general localities sampled in this study. The inset map of western North America depicts the Great Basin Desert (shaded area) as defined using floristic data from Cronquist *et al.* (1972). In both maps, the outline of the state of Nevada is shown for orientation.

Pallid kangaroo mice were sampled from 27 general localities throughout the species’ geographical range in the Great Basin Desert (Fig. 1). A total of 98 specimens were used in this molecular study: 95 specimens were collected in the wild between 1999 and 2005, and three specimens (all from Alamo) were collected in 1975 (see Appendix). Mitochondrial DNA sequence data from two specimens from Goldfield (Appendix) were taken from Hafner *et al.* (2006): GenBank accession numbers for 16S and *Cytb* (includes a small, adjoining section of tRNA^Glu^) are DQ422910, DQ422911 and DQ422937, DQ422938, respectively.

Following Hafner *et al.* (2006), initial outgroup taxa included the sister species, the dark kangaroo mouse (*M. megacephalus*), representative kangaroo rats, the chisel-toothed kangaroo rat (*Dipodomys microps* Merriam) and the desert kangaroo rat (*D. deserti* Stephens), and a pocket mouse, the little pocket mouse (*Perognathus longimembris* Coues). Final selection of outgroup taxa (Appendix) excluded *P. longimembris* because our preliminary analyses and previous phylogenetic analyses showed it to be less closely related to *Microdipodops* than is *Dipodomys*. Our outgroup selection was also supported by other studies (Hafner, 1982; Hafner & Hafner, 1983; Rogers, 1990; Hafner, 1993; Mantooth *et al.*, 2000; Alexander & Riddle, 2005; Hafner *et al.*, 2006, 2007). Sequence data from two outgroup specimens (*D. microps* and *M. megacephalus*) in the Appendix were taken from Hafner *et al.* (2006): GenBank accession numbers for 16S and *Cytb* (includes a small section of tRNA^Glu^) are DQ422887, DQ422914 and DQ422895, DQ422917, respectively. Animals collected during the course of this study were treated in a humane manner following procedures approved by the American Society of Mammalogists (Gannon *et al.*, 2007) and Occidental College’s Institutional Animal Care and Use Committee.

### Mitochondrial DNA analyses

Portions of two mitochondrial genes, 16S and *Cytb*, were selected for analysis in this study because of their contrasting evolutionary rates (16S is more conservative than *Cytb*; Ferris *et al.*, 1983; Springer *et al.*, 2001; Hafner *et al.*, 2006, 2007). Different rates of molecular change should facilitate the resolution of clades at both deep (16S) and shallow (*Cytb*) temporal levels, and allow for more detailed phylogenetic inference (Hillis & Dixon, 1991; Meyer, 1994). Laboratory procedures pertaining to DNA extraction, mitochondrial DNA (mtDNA) amplification, purification and sequencing were conducted as described by Hafner *et al.* (2006). Amplifications of 16S and *Cytb* were optimized using the following thermal profile: initial denaturation at 95°C (30 s), followed by 35 cycles of denaturation at 95°C (30 s), annealing at 52°C (60 s), and extension at 72°C (90 s), and a final extension at 72°C for 5 min. Polymerase chain reaction (PCR) and sequencing of the 16S gene were performed using 16Sar and 16Sbr human primers (Palumbi, 1996). The *Cytb* gene was amplified and sequenced using the primers MVZ05 and MVZ04 (Smith & Patton, 1991), which were placed in conserved regions of the 5′ adjacent tRNA^Glu^ and *Cytb* gene, respectively. Regular sequencing yielded a continuous section that includes a small (40 base pairs, bp) portion of tRNA^Glu^, non-coding bases, and 403 bp of the protein-coding *Cytb* gene. This continuous section of tRNA^Glu^ and *Cytb* was considered only in the phylogenetic analysis of the combined data set (16S + *Cytb* + tRNA^Glu^), not in the independent *Cytb* analyses.

Double-stranded sequences (light and heavy strands) for each individual were edited and assembled in GeneTool 1.0 (Biotools, Inc., Edmonton, Canada). All new sequences of *M. pallidus* (*n*=96) were submitted to GenBank (GenBank accession numbers DQ534206–DQ534301 for 16S; DQ534302–DQ534397 for *Cytb*, includes tRNA^Glu^). New sequence data for additional outgroup specimens were also submitted to GenBank: two specimens of *M. megacephalus* (GenBank accession numbers DQ870281, DQ870313 for 16S; DQ870362, DQ870405 for *Cytb*, includes tRNA^Glu^) and a specimen of *D. deserti* (GenBank accession number DQ870428 for 16S; DQ870429 for *Cytb*, includes tRNA^Glu^). Multiple sequence alignments were performed using ClustalX (Thompson *et al.*, 1997) with the default settings (gap opening=10, gap extension=0.20) for 16S, *Cytb* and the combined (16S + *Cytb* + tRNA^Glu^) data set. All alignments were examined visually and edited manually in MacClade 4.0 (Maddison & Maddison, 2000), with unambiguous alignment at all positions allowing for postulated gaps to be verified without the use of structural models (Leaché & Reeder, 2002; Carranza *et al.*, 2006). Unique haplotypes were identified using Arlequin 3.01 (Excoffier *et al.*, 2005) and all subsequent analyses were based on unique haplotypes. mega 3.1 (Kumar *et al.*, 2004) was used to calculate transition/transversion ratios, estimate base composition and test our data sets for saturation.

Phylogenetic analyses were first performed separately on the 16S (543 bp) and *Cytb* (403 bp) data sets, then on the combined (16S + *Cytb* + tRNA^Glu^) alignment of 991 bp, to identify possible incongruence between the gene fragments (Wiens, 1998; Leaché & Reeder, 2002; Townsend *et al.*, 2004). The partition homogeneity test (PHT; Farris *et al.*, 1994) was implemented in paup* 4.0b10 (Swofford, 2003) to further determine phylogenetic congruence. Executed under maximum-parsimony settings, the PHT was run with 1000 partition replicates, 10 random taxon-additions per replicate, and no more than 500 equally most parsimonious trees retained per replicate to limit computation times. A non-significant PHT result (*P*=0.89) allowed for combination of the three mtDNA gene fragments. Maximum parsimony and neighbour-joining methods (paup* 4.0b10) were used subsequently to analyse each of our three mtDNA data sets (16S, *Cytb* and combined), and all trees were virtually identical topologically except for minor changes within the terminal branches. Further analysis of our combined data set was conducted using maximum-likelihood approaches (paup* 4.0b10), as well as Bayesian methods (MrBayes 2.01; Huelsenbeck & Ronquist, 2001).

Maximum-parsimony analyses were conducted with the following settings: full heuristic searches of equally weighted sites, simple sequence addition, tree bisection–reconnection branch swapping, and multiple parsimonious trees saved. Nodal support for the maximum-parsimony consensus tree was evaluated by calculating 1000 bootstrap pseudoreplicates (Felsenstein, 1985) using paup* 4.0b10. Bremer support values (Bremer, 1994) were obtained using both paup* 4.0b10 and TreeRot (ver. 2; Sorenson, 1999). paup* 4.0b10 was also used to determine the consistency index (CI) and retention index (RI) and to test for the presence of phylogenetic signal (Hillis & Huelsenbeck, 1992).

Estimates of percentage nucleotide sequence divergence were calculated in mega 3.1 for each gene fragment and the combined data set using uncorrected *p* distance and the pairwise-deletion option (gaps removed pairwise per comparison). For comparison purposes, genetic distances were also calculated using Kimura’s two-parameter model (Kimura, 1980). Uncorrected *p* distance was used to perform neighbour-joining analyses (Nei & Kumar, 2000). Neighbour-joining distance trees were bootstrapped with 1000 pseudoreplicates to assess clade reliability.

The most appropriate model of nucleotide evolution for the combined data set, as suggested by ModelTest (ver. 3.7; Posada & Crandall, 1998), was the general time-reversible model with invariant sites and among-site variation (GTR + *I* + Γ; Yang, 1994; Gu *et al.*, 1995). This model of evolution, determined under the Akaike information criterion (Johnson & Omland, 2004; Posada & Buckley, 2004), was also used to compare rates of nucleotide substitution with the molecular clock. Maximum-likelihood analyses were conducted using the parameters specified by ModelTest and a full heuristic search under maximum-parsimony settings. A full heuristic bootstrap (200 pseudoreplicates) was then performed on the constructed maximum-likelihood tree.

Bayesian phylogenetic analyses were performed in MrBayes 2.01 using GTR + *I* + Γ, with the specific model parameters treated as unknowns with uniform priors and estimated by each Bayesian analysis (Leaché & Reeder, 2002). Four incrementally heated chains (Metropolis-coupled Markov chain Monte Carlo; Huelsenbeck & Ronquist, 2001) were run concurrently for 10,000,000 generations and were sampled every 1000 generations. These 10,000 data points were acquired twice in independent Bayesian analyses to make sure the searches were not limited to local optima (Leaché & Reeder, 2002). Stationarity was evaluated graphically by plotting log-likelihood values of sample points against generation time, then eliminating the first 200 trees prior to stationarity as burn-in values. The remaining 9800 equilibrium trees from each independent analysis were used to create a 50% majority-rule consensus tree, where each clade’s posterior probability value is indicative of the percentage of samples that recover that particular clade (Huelsenbeck & Ronquist, 2001).

### Directional analyses of phylogeographical patterns

Compass orientations between pairs of localities whose individuals share haplotypes represent fine-scale phylogeographical patterns and may provide insights regarding historical trends in gene exchange and movement patterns of kangaroo mice. Directional analyses of phylogeographical patterns (DAPP), a novel approach presented here, relies on axial data (angular measurements of undirected lines, 180° ambiguity) that were measured between all combinations of pairwise localities involved in haplotype sharing among individuals of kangaroo mice. Angular measurements were recorded to the nearest 1° with the aid of a 360° ruler on distribution maps of *M. pallidus*. Angular data were reduced and the mean vector (μ) was calculated for each major geographical unit, as well as the pooled sample of *M. pallidus*. Several uniformity tests were conducted to determine if each sample of orientations between pairwise localities was distributed in a random (isotropic) manner: Rayleigh’s uniformity test, Rao’s spacing test and Kuiper’s test (Batschelet, 1981; Fisher, 1993; Kovach, 2006). The Mardia–Watson–Wheeler test and the Watson *U*^2^ test were used to test whether two samples have the same angular distribution. Oriana software (Kovach, 2006) was used to calculate all circular statistics involved in DAPP.

## Results

### Field work and geographical distribution

Collecting activities for this study, involving 13,900 trapnights and resulting in the trapping of 128 individuals of *M. pallidus*, yielded an overall trapping success of 0.92% for *M. pallidus*. Despite setting traps at known localities (Hall, 1941; Hafner, 1981) or at new sites in habitats judged (by J.C.H.) to be appropriate for this species, trapping results show that *M. pallidus* is among the least abundant of the nocturnal desert rodents in sandy habitats of the Great Basin (data available on request). Considering only those localities where individuals of *M. pallidus* were captured, the mean trapping success was only 2.88%; the range in trapping success was 0.25% (one capture from 400 trapnights) to 14.0% (14 captures from 100 trapnights).

We note several adjustments to Hall’s (1941) portrayal of the geographical distribution of *M. pallidus* in the north-western, south-central and north-eastern portions of the species’ distribution (Fig. 1). Specifically, kangaroo mice around the southern end of Pyramid Lake (the general localities of Nixon and Wadsworth) are identified as *M. pallidus* not *M. megacephalus* (cf. Hall, 1941, 1946; Mantooth *et al.*, 2000); this finding corroborates Hafner (1981) and extends the north-western distribution margin *c.* 50 km. In the south-central portion of the geographical range, the locality of SE Goldfield (at Stonewall Flat) extends the known range of *M. pallidus* southward about 30 km; prior to this study, SE Tonopah (=‘north shore of Mud Lake’, Hall, 1941: 272) represented the southernmost central locality. Lastly, the localities of Currant and NE Warm Springs extend the north-eastern distributional arm of the species about 30 km to the north and west from the locality of Lockes (referred to as ‘Locks Ranch’ by Hall, 1941: 273). The presence of *M. pallidus* at the NE Warm Springs locality was also reported by Hafner (1981) and Hafner *et al.* (1996).

### Mitochondrial DNA sequence characteristics

The combined (16S + *Cytb* + tRNA^Glu^) data set, including all unique haplotypes of *M. pallidus* and outgroup taxa, shows a total of 238 variable characters (96, 126 and six variable characters, respectively). Rates of nucleotide substitution are in accordance with a molecular clock model (using the combined data set, χ^2^ = 43.35, *P*>0.05 with samples of *M. megacephalus* designated as the outgroup, and χ^2^ = 29.67, *P*>0.05 with species of *Dipodomys* designated as outgroup taxa). Mean base frequencies for A, C, G and T are 0.321, 0.243, 0.168 and 0.269, respectively (0.337, 0.209, 0.195 and 0.260, respectively for 16S and 0.287, 0.294, 0.140 and 0.279, respectively for *Cytb*; data for tRNA^Glu^ available on request). Chi-square tests for possible heterogeneity of base frequencies across all samples are not significant for the combined data set (χ^2^ = 8.779, *P*=1.000) nor for each gene (χ^2^ = 2.835, *P*=1.000 for 16S; χ^2^ = 8.692, *P*=1.000 for *Cytb*); hence, it is doubtful that base compositional heterogeneity causes phylogenetic bias. Mean base frequencies for A, C, G and T for unique *M. pallidus* haplotypes only are 0.338, 0.209, 0.194 and 0.259, respectively, for 16S and 0.287, 0.294, 0.140 and 0.279, respectively, for *Cytb*.

Transition/transversion ratios for 16S, *Cytb* and the combined data set are 2.737, 3.327 and 3.377, respectively (over all positions, using uncorrected *p*, and with only samples of *M. pallidus*). Following the methods of Barker & Lanyon (2000), plots of number of transitions vs. uncorrected *p* distance show no evidence for saturation for 16S nor for *Cytb* for the unique haplotypes of *Microdipodops* studied. However, third-position transitions for *Cytb* show saturation when *D. deserti* and *D. microps* are included in the analyses. Tests for phylogenetic signal in our data sets (over all unique haplotypes and with both species of *Dipodomys* designated as outgroups) show significance for 16S (96 variable characters, 29 haplotypes, *g*_1_ = –0.697, *P*<0.01) and for *Cytb* (126 variable characters, 31 haplotypes, *g*_1_ = –0.463, *P*<0.01).

### Haplotypic variation in *M. pallidus*

A total of 42 unique composite haplotypes and 87 polymorphic sites are identified from the combined mtDNA data set that includes 98 individuals of *M. pallidus* from 27 general localities. Considering 16S and *Cytb* separately, there are 24 and 26 unique haplotypes and 39 and 46 polymorphic sites, respectively, for these genes.

Twenty of 27 general localities are represented by multiple individuals and, hence, are available for an assessment of intrapopulational mitochondrial sequence variation (San Antonio is excluded here because it is identified as a locality of contact between divergent haplotypes; see beyond). There is a mean (and range) of 4.45 (2–10) individuals sampled per locality for these 20 localities. Patterns of within-population variation are similar for 16S and *Cytb*. For example, the mean number of haplotypes per locality is 1.95 and 1.90 for 16S and *Cytb*, respectively. For 16S, there is no significant functional relationship between the number of haplotypes and sample size seen at a locality (*b*=0.108; *P*=0.280). However, a significant linear trend between the number of haplotypes and sample size per locality is evident for *Cytb* (*b*=0.225; *P*=0.013). Lastly, the mean number of polymorphic sites per population is 1.40 and 1.20 for 16S and *Cytb*, respectively.

### Phylogenetic analyses

**Figure 2:**
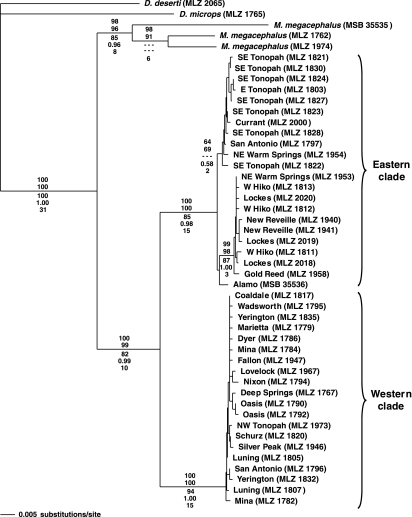
Distance (neighbour-joining) tree based on the composite mtDNA sequence data and showing the relationships among the 42 unique haplotypes of *Microdipodops pallidus* Merriam. Distance and parsimony bootstrap support values are indicated above the nodes, with maximum-likelihood support values, Bayesian posterior probabilities and Bremer decay indices below the nodes.

Analysis of the combined (991 bp) mtDNA sequence data for the 42 ingroup haplotypes of *M. pallidus* and the five outgroup species yields 165 characters that are parsimony-informative (66, 90 and four parsimony-informative characters for the separate 16S, *Cytb*, and tRNA^Glu^, respectively). Parsimony analysis for the combined data set shows 132 most parsimonious trees (CI=0.732; RI=0.910). Phylogenetic analyses using maximum-parsimony, neighbour-joining, maximum-likelihood and Bayesian approaches produce trees that are virtually identical in topology; only slight differences in the placement of *M. pallidus* haplotypes at extreme terminal branches are evident. Monophyly of the genus *Microdipodops* is supported strongly in all analyses, and all analyses show that the 42 unique haplotypes of *M. pallidus* form a clear sister clade relative to the samples of *M. megacephalus* (Fig. 2).

**Figure 3:**
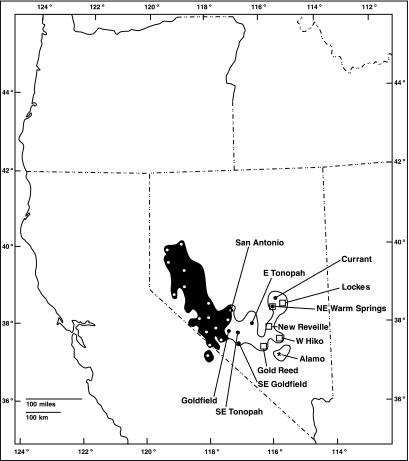
Distribution map of the eastern and western clades of *Microdipodops pallidus* Merriam. Each of the two phylogroups is represented by a main distributional body and a southern peripheral isolate. Note that both principal haplotypes are sympatric at San Antonio. Within the eastern clade, three subunits are recognized: south-central (dots), eastern (squares) and south-eastern (star) subunits. Haplotypes from both south-central and eastern subunits co-occur at NE Warm Springs.

Support is also very high for the recognition of two basal clades within the currently recognized species *M. pallidus*: an eastern clade and a western clade (Figs 2 & 3). These clades are distributed parapatrically, except at the San Antonio locality (Fig. 3), where both eastern and western haplotypes are found in sympatry. Of three kangaroo mice examined from San Antonio, one individual represents an eastern haplotype and two individuals are aligned with the western clade.

The western clade shows very little structure and appears to represent a large polytomy (Fig. 2). Although there is little mtDNA differentiation among haplotypes in the western clade (one or two base substitutions), the distributional isolate from the Deep Springs locality forms a weakly resolved clade with the Oasis locality (Figs 1 & 2). Nodal support values for the distance, maximum-parsimony, maximum-likelihood, Bayesian and Bremer analyses are 54, 60, 71, 0.97 and 1, respectively, for this clade.

Haplotypes of the eastern clade seem to assort imperfectly into three geographical subunits: a south-central subunit, an eastern subunit, and a south-eastern peripheral isolate (Figs 2 & 3). The south-central subunit is resolved weakly, and includes haplotypes from seven localities: SE Tonopah, E Tonopah, Currant, San Antonio, NE Warm Springs, Goldfield and SE Goldfield (the latter two localities are not shown in Fig. 2). The eastern subunit is a well supported clade and includes haplotypes from five localities aligned in a north–south distributional prong: NE Warm Springs, W Hiko, Lockes, New Reveille and Gold Reed (Fig. 2). Lastly, the isolated population near Groom Lake (Alamo) appears to represent a distinct matrilineage. Note that our phylogenetic analyses place haplotypes from Currant (two haplotypes, *n*=5) in the south-central subunit despite Currant’s geographical position at the northern tip of the eastern distributional prong (Fig. 3). Moreover, haplotypes from NE Warm Springs (five haplotypes, *n*=5) are represented in both the south-central subunit (four haplotypes) and the eastern subunit (one haplotype); NE Warm Springs, like Currant, is located at the northern tip of the eastern subunit (Fig. 3).

As expected from its known higher rate of substitution, percentage divergence values for *Cytb* within and among *Microdipodops* clades are routinely larger than corresponding values for 16S (Table 1). For the gene fragments examined here, *Cytb* divergence values at the deeper nodes (e.g. between eastern vs. western clades of *M. pallidus* and the node for the species of kangaroo mice) are approximately twice those of 16S. Eastern and western clades of *M. pallidus* are distinguished by high levels of sequence divergence (*c.* 8% sequence divergence for *Cytb*), as are the currently recognized species of *Microdipodops* (*c.* 13–15% for *Cytb*; Table 1). The peripheral isolate in the western clade (Deep Springs) shows only minimal divergence from other western populations, but the peripheral isolate in the eastern clade, Alamo, is modestly divergent from adjacent eastern populations (Table 1; Fig. 3).

**Table 1:** Mean pairwise sequence divergence values within and among selected clades of *Microdipodops* examined in this study.

Comparison	16S	*Cytb*	All
*Microdipodops pallidus* contrasts			
Western clade			
Within western clade	0.28 (0.28)	0.68 (0.69)	0.32 (0.32)
Deep Springs isolate vs. other western clade	0.32 (0.32)	0.60 (0.60)	0.35 (0.36)
Eastern clade			
Within eastern clade	0.76 (0.77)	1.04 (1.05)	0.70 (0.70)
South-central subunit vs. eastern subunit	1.01 (1.02)	1.43 (1.44)	1.02 (1.03)
South-central subunit vs. Alamo isolate	0.95 (0.97)	1.24 (1.25)	0.97 (0.98)
Eastern subunit vs. Alamo isolate	1.11 (1.13)	1.18 (1.19)	1.05 (1.05)
Eastern clade vs. western clade	3.99 (4.12)	7.50 (8.01)	5.20 (5.43)
*M*. *pallidus* vs. *M*. *megacephalus*	6.13 (6.40)	13.21 (14.83)	9.61 (10.36)

Mean percentage divergence estimates for both uncorrected *p* distance and Kimura’s two-parameter model (in parentheses) are given for individual genes and the combined data set (All).

### Haplotype sharing and orientation analyses

**Figure 4:**
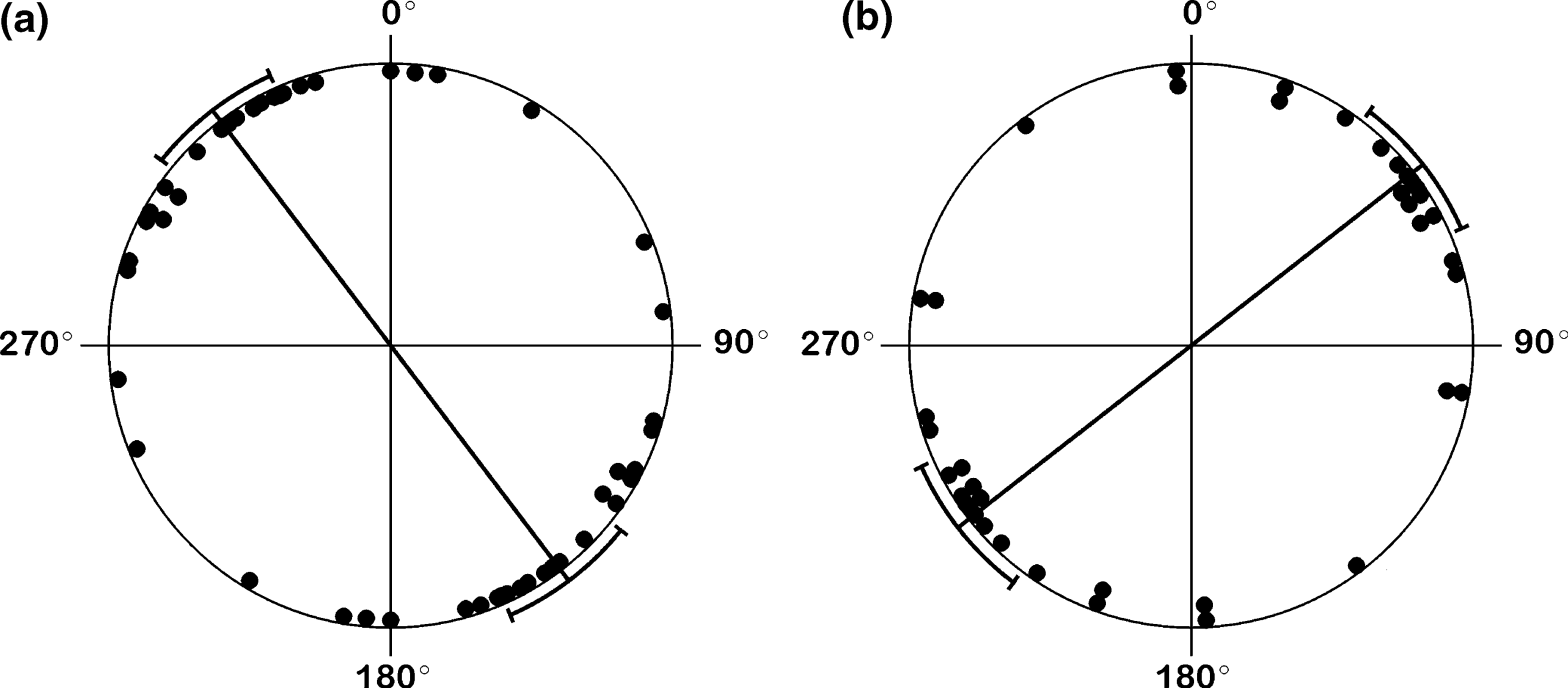
Angular trends derived from orientation analyses of haplotype-sharing patterns between pairs of localities of *Microdipodops pallidus* Merriam. The western (a) and eastern (b) clades show significantly different bidirectional axial patterns over geography (mean orientations and 95% confidence intervals are indicated). The north-west to south-east orientation in the western clade (a) and the north-east to south-west directional pattern in the eastern clade (b) obtained from DAPP (see text) signal different histories of gene flow in the two clades.

Nine of 42 (21.4%) of the unique composite haplotypes identified in Fig. 2 are present at two or more general localities (Table 2). There are 25 and 20 pairwise combinations of haplotype sharing between localities in the western clade and eastern clade of *M. pallidus*, respectively (Fig. 4), yielding a total of 45 possible pairwise combinations of axial data that are available for DAPP analysis. Orientation data based on haplotype sharing for all pairwise localities of *M. pallidus* show no departure from a uniform distribution (Rayleigh’s *Z*=0.141, *P*=0.87; Rao’s *U*=138, *P*>0.10; Kuiper’s *V*=0.997, *P*>0.15). However, when the axial data are examined separately for the western and eastern clades of *M. pallidus*, clear orientation patterns emerge from the DAPP (Fig. 4). For the western clade (*n*=25), the mean vector μ = 142.751° (and also 322.751° because of the bidirectional or axial nature of the data) and tests for uniformity are all significant (Rayleigh’s *Z*=7.332, *P*<0.001; Rao’s *U*=161.2, *P*<0.05; Kuiper’s *V*=2.278, *P*<0.01). Angular data for the eastern clade (*n*=20) show a mean vector μ = 51.862° (and 231.862°) and also depart significantly from uniformity (Rayleigh’s *Z*=6.104, *P*=0.002; Rao’s *U*=192, *P*<0.01; Kuiper’s *V*=2.08, *P*<0.01). Hence, haplotype sharing between pairwise localities shows a distinct (non-random) north-west to south-east orientation in the western clade and a distinct north-east to south-west directional pattern in the eastern clade (Fig. 4).

**Table 2:** Sharing of unique composite haplotypes of *Microdipodops pallidus* over geography.

Unique haplotype	Number of localities	Distribution
Coaldale MLZ 1817	7	Western Clade: Coaldale (MLZ 1817), Dyer (MLZ 1785, MLZ 1787, and MLZ 1789), Luning(MLZ 1810), Marietta (MLZ 1777 and MLZ 1778), Mina (MLZ 1780, MLZ 1781, and MLZ1783), Schurz (MLZ 1819) and Silver Peak (MLZ 1945)
SE Tonopah MLZ 1823	5	Eastern Clade: E Tonopah (MLZ 1823, MLZ 1825 and MLZ 1826), E Tonopah (MLZ 1801 andMLZ 1802) Goldfield (MLZ 1746), NE Warm Springs (MLZ 1955) and SE Goldfield (MLZ2051)
SE Tonopah MLZ 1830	4	Eastern Clade: SE Tonopah (MLZ 1830), Currant (MLZ 2001 and MLZ 2004), Goldfield(MLZ 1743) and NE Warm Springs (MLZ 1952)
Luning MLZ 1805	3	Western Clade: Luning (MLZ 1805, MLZ 1806 and MLZ 1809), San Antonio (MLZ 1798)and Schurz (MLZ 1818)
SE Tonopah MLZ 1824	2	Eastern Clade: SE Tonopah (MLZ 1824) and SE Goldfield (MLZ 2052)
SE Tonopah MLZ 1828	2	Eastern Clade: SE Tonopah (MLZ 1828 and MLZ 1829) and E Tonopah (MLZ 1804
Currant MLZ 2000	2	Eastern Clade: Currant (MLZ 2000, MLZ 2002, and MLZ 2003) and NE Warm Springs(MLZ 1906)
NE Warm Springs MLZ 1953	2	Eastern Clade: NE Warm Springs (MLZ 1953) and Lockes (MLZ 2017)
San Antonio MLZ 1796	2	Western Clade: San Antonio (MLZ 1796) and Yerington (MLZ 1833, MLZ 1836, MLZ 1837,and MLZ 1839)

Nine unique haplotypes, identified in Fig. 2, are present at two or more general localities and are available for directional analyses of phylogeographical patterns (DAPP; see text). In total, there are 45 pairwise combinations of shared haplotypes (25 in the Western Clade and 20 in the Eastern Clade) that provide the basis for directional data.

## Discussion

### Abundance of kangaroo mice

Although the reporting of measures of relative abundance (e.g. percentage trap success or capture rate) is not traditional practice in systematic and biogeographical studies, such information is useful to future field biologists, conservationists and wildlife managers who are interested in monitoring the viabilities of populations over time. In a very simple way, the routine reporting of collecting techniques and measures of abundance in systematic and phylogeographical studies may aid in strengthening the intellectual linkage between phylogeography and conservation biology, and we encourage future workers to report these kinds of data. The present conservation status (International Union for Conservation of Nature Red List Category) of *M. pallidus* is ‘lower risk, least concern’ (Hafner & Hafner, 1998: 80; includes one vulnerable subspecies) and the species is protected in both California and Nevada. Future application of the Mace & Lande (1991) criteria for assessing the conservation status of kangaroo mice requires data pertaining to abundance, especially changing abundance. Data on abundance are important for understanding the conservation status of all species, but these data seem particularly important for those kinds of organism that are considered to be rare in nature.

Hall (1941, 1946) remarked that naturalists considered kangaroo mice to be rare. However, there is very little information in the literature that pertains to estimates of abundance of kangaroo mice, especially for *M. pallidus*. Despite special efforts to collect *M. pallidus* in suitable habitats across its geographical distribution, our general experience is that the pallid kangaroo mouse is a rare member of the nocturnal desert rodent community. Inspection of trapping data in our field notes (data available on request) reveals that *M. pallidus* falls routinely in the rare-species category that predominates the classic ‘hollow curve’ of number of species vs. species abundance in community ecology (Krebs, 1994; McGill, 2006). In our experience in sandy habitats of the Great Basin, usually one or two species of kangaroo rat are numerically dominant, followed by three to five less-common species; kangaroo mice are invariably among the last species to be recorded in our notebooks when checking traps. Percentage trapping success for *M. pallidus* is usually an order of magnitude smaller than percentage trapping success for the one or two abundant species that we often encounter in communities of nocturnal desert rodents in the Great Basin (data available on request).

### Geographical distribution, ecology and conservation biology

Hall (1941) reported an elevational range for *M. pallidus* of 1189–1737 m (3900–5700 ft), and emphasized that this species occurs in habitats above those that support the creosote bush, *Larrea* Cavanilles, and below those that support sagebrush, *Artemisia*. In our experience, *M. pallidus* is found most frequently in floral communities where greasewood, *Sarcobatus* Nees von Esenbeck, and saltbush, *Atriplex* Linnaeus, predominate. The present study confirms the lower elevational and floral limits reported by Hall (1941); *M. pallidus* has never been captured in habitats associated with the Lower Sonoran Life-Zone, and these rodents are found only at their lower elevational extreme in the northern portion of their distribution (Soda Lake: Hall, 1941; Fallon: this study). Hafner *et al.* (1996) extended the upper elevational limit of the species to 1829 m (6000 ft), and this record is affirmed in this study (captures at both NE Warm Springs and Currant). Importantly, at this upper elevational margin, *M. pallidus* was captured within a few metres of *Artemisia* bushes at NE Warm Springs and at Currant. At both these high-elevation localities, *M. megacephalus* was captured in the same trap lines that yielded *M. pallidus*. As noted by Hall (1941), *M. pallidus* occurs on fine, sandy soils supporting vegetation. At every place where we captured *M. pallidus* except one (SE Goldfield), the soil was fine, deep sand with little or no gravel overlay. At SE Goldfield (at the southern margin of the species’ range), *M. pallidus* was taken in fine, deep, sandy soil that had an unusually heavy overlay of large-sized (> 10 mm) gravel. In contrast to *M. pallidus*, *M. megacephalus* is usually found on sandy soils with a gravel overlay, at higher elevations, and in habitats dominated by *Artemisia* and/or rabbit brush, *Chrysothamnus* Nuttall. Differences in habitat affinity between the species of kangaroo mice were evaluated by Hafner *et al.* (1996) and their conclusions support the habitat differences for *M. pallidus* and *M. megacephalus* described here.

Three-quarters of a century after Hall’s (1941) field work on *M. pallidus*, we document a geographical distribution for the species that is remarkably unchanged. This finding is particularly noteworthy against the backdrop of recent concerns over global warming, documented changes in species distributions, and the positive bias in the literature (Parmesan *et al.*, 1999; Beever *et al.*, 2003; Parmesan & Yohe, 2003; Wagner *et al.*, 2003; Perry *et al.*, 2005). The few minor modifications in our portrayal of the geographical range of *M. pallidus* noted in this study indicate no evidence for any natural, systematic distributional changes. What may appear as a northern range expansion near the southern end of Pyramid Lake (localities of Nixon and Wadsworth; Fig. 1) is actually due to the reidentification of specimens collected from that region. The localities of Currant and NE Warm Springs extend the north-eastern distributional arm of *M. pallidus* northward (and elevationally upward) as compared with Hall’s (1941) understanding of the species’ distribution. However, neither Hall nor members of his field party visited this remote area (Hall, 1946) and, therefore, the question of a possible natural range adjustment over the ensuing years is moot.

After repeated efforts to collect *M. pallidus* at and near its type locality, Mountain Well (Churchill County, *c*. 35 km east of our Fallon locality, see Fig. 1), we conclude that kangaroo mice are probably locally extinct in this area. There are two other instances where we failed to collect *M. pallidus* from historical sites: Sand Mountain, Churchill County (‘37 km south-east Fallon’; Brown, 1973: 777) and Tikaboo Valley, Lincoln County (‘eight miles southwest of Hancock Summit’; Hall, 1941: 274; this is *c*. 10–15 km east of our Alamo locality in Emigrant Valley; Fig. 1). Exhaustive trapping was not conducted at Sand Mountain and, therefore, we cannot state that this population is not extant. However, exhaustive trapping in Tikaboo Valley permits us to conclude that kangaroo mice are most likely to be locally extinct in Tikaboo Valley. Although Mountain Well is in the northern portion and Tikaboo Valley is in the southern portion of the species’ distributional range, the two areas are similar to the extent that they both harbour small, undisturbed patches of habitat that seem appropriate for *M. pallidus* but, for unknown reasons, kangaroo mice do not occur now in either of these areas.

We agree with Hall (1941) in recognizing two southern distributional isolates for *M. pallidus*: Deep Springs Valley (our locality of Deep Springs), and Emigrant and Tikaboo Valley areas east of Groom Lake (our Alamo locality from Emigrant Valley; Fig. 1). Kangaroo mice from Deep Springs Valley, although only minimally distinct genetically from other populations in the western clade (Fig. 2; Table 1) are isolated geographically from other *M. pallidus* populations (e.g. Oasis) by a rather dramatic ridge of mountains at the southern terminus of the White Mountains (the Gilbert Pass region). Given the valley’s small size and isolation, it is not surprising that all 10 animals from Deep Springs are fixed for the same unique haplotype. Given the present extent of livestock grazing and invasive plants in Deep Springs Valley, we suggest that the population of kangaroo mice in Deep Springs Valley be monitored closely to ensure the long-term welfare of this population.

The peripheral isolate from the Emigrant and Tikaboo Valley areas (represented by Alamo on Fig. 1) appears to be a distinct haplotypic lineage of the eastern clade (Fig. 2; Table 1) and is isolated physiographically from all other populations of kangaroo mice to the west and north by the Belted Range, Chalk Mountain, and the Groom Range. Based on morphology, Hall (1941) recognized pale kangaroo mice from the areas east of Groom Lake (in Emigrant Valley and Tikaboo Valley) as a distinct subspecies, *M. p. purus*. Given the phylogeographical and taxonomic importance of this taxon, it is especially important in the context of conservation biology to document its presence and viability today. Unfortunately, access is restricted in the militarily sensitive area of Groom Lake in Emigrant Valley, and kangaroo mice seem no longer to exist in Tikaboo Valley. The samples used in this study from Emigrant Valley were obtained in 1975, before the expansion of the existing boundary of the military range. Unfortunately, no samples of kangaroo mice from this region have been obtained in the ensuing three decades and the conservation status of these kangaroo mice is unknown.

Fire, livestock grazing, invasive plants and agriculture represent the possible ‘big four’ threat factors with regard to kangaroo mouse habitat. Of the big four, habitat loss associated with agricultural practices (especially alfalfa farming) seems to be the most serious concern for *M. pallidus*, which often occurs in the valley floors where the growth of alfalfa is favoured. In our experience, areas of concern due to expanding agriculture include Lahontan Valley (Fallon), Mason Valley (Yerington), Fish Lake Valley (Oasis and Dyer), and Sand Spring Valley (W Hiko, recognized as Penoyer Valley from Hall, 1941). Wild fires are always an imminent threat throughout the Great Basin but, fortunately, destruction of habitat by wild fires (and the subsequent invasion of introduced weed species) has not been a main factor affecting the distribution and abundance of *M. pallidus*. Livestock (mainly cattle) grazing, common throughout most of the distribution of *M. pallidus* since the 1860s (Wagner *et al.*, 2003), seems to be tolerated by *M. pallidus* in most places. At present, most of the localities of *M. pallidus* are still remarkably free or largely free of invasive plants (e.g. Russian thistle, *Salsola* Linnaeus and cheat grass, *Bromus* Linnaeus) common elsewhere in the Great Basin.

### Phyletic patterns and historical biogeography

The molecular data (Figs 2 & 3) identify eastern and western clades of *M. pallidus*, each represented by a principal distributional body and a peripheral isolate. The geographical distributions of the eastern and western clades are approximately equal in size and show nearly the same number of unique haplotypes (22 and 20, respectively). The western clade of *M. pallidus* is distributed in the Lahontan Trough (Reveal, 1979), a low-elevation, north-west-trending region that is part of a geologically complex area known as the Walker Fault Zone (also termed the Walker Belt or Walker Lane: Fiero, 1986; Morrison, 1991; Grayson, 1993; Hafner *et al.*, 2006). Little phylogenetic structure is evident in the western clade, such that a comb-like pattern of relationships emerges (Figs 1 & 2). Reveal (1979) suggested that the Lahontan Trough represented a corridor for the northward range expansion of biota following the Pleistocene. The comb-like pattern of relationships is consistent with a hypothesis of rapid range expansion of kangaroo mice in the Lahontan Trough. However, instead of considering the trough as a unidirectional corridor for northward range expansion since the Pleistocene, we view the Lahontan Trough as a corridor that allowed repeatedly northward and southward distributional range adjustments of *M. pallidus* in response to climatic changes throughout the Pleistocene. The presence of slightly more differentiated haplotypes in the southern portion of the western clade (Fig. 2) indicates that this area (or regions farther south) may have served as a refugium during pluvial maxima. In contrast, it is likely that the distribution of *M. pallidus* in the north was dictated by the waxing and waning of ancient Lake Lahonton (i.e., near existing Pyramid Lake and Walker Lake).

The geographical range of the eastern clade is bounded to the south by the Mojave Desert and to the north by the southern end of the Toiyabe, Taquima, Monitor, Hot Creek, Pancake and Quinn Canyon Ranges. The three subunits of the eastern clade (Fig. 3) appear to be separated physiographically from one another: the Hot Creek and the Kawich Ranges lie between the south-central subunit and the eastern subunit; the Belted Range, Chalk Mountain and the Groom Range separate the eastern subunit from the south-eastern peripheral isolate (Alamo). Six of the seven haplotypes recorded from the localities of Currant and NE Warm Springs do not share affinity with other populations of the eastern subunit but, instead, are genetically more closely related to populations from the south-central subunit (Fig. 3). Presumably, these disjunct haplotypes from Currant and NE Warm Springs represent relictual populations of a once more broadly distributed south-central subunit that was able to flank the southern ends of the Hot Creek and Kawich Ranges and gain access to the sandy habitats to the east and north-east.

The south-eastern peripheral isolate (Alamo) of the eastern clade is genetically distinct from the other eastern subunits (*c*. 1% sequence divergence; Table 1). All three specimens available from this population share the identical haplotype, as might be expected for a small, distributional isolate. Although this isolate is adjacent to the eastern subunit, our mtDNA sequence data do not show a sister-clade relationship with that subunit and, indeed, are unable to resolve the relationships among the three subunits.

### Directional analysis of phylogeographical patterns

Historical routes of gene exchange may be detected by angular analyses of haplotype sharing between pairwise localities. The specific, quantitative routes documented in this study (a north-west to south-east orientation in the western clade and a north-east to south-west directional pattern in the eastern clade; Fig. 4) allow us to make two observations. First, the angular trends are consistent with an interpretation that populations of kangaroo mice adjusted their distributions in predominantly northward and southward directions in response to past climatic shifts of warming and cooling of the Pleistocene. Second, the intersection of these two orientation trends in the vicinity of southern Nevada suggests that this area may have represented a broad, refugial region for kangaroo mice at the height of pluvial periods. Unfortunately, the age of these haplotype-sharing patterns is not known at this time.

The telltale signs of historical patterns of gene flow reflected in DAPP are, of course, constrained by mountain ranges and the availability of appropriate sandy habitats. Although it is tempting to assume that one may infer orientation patterns of gene flow from simply a casual inspection of a distribution map, we urge caution in making this assumption without knowledge of actual orientation data from genetic patterns. In addition, we note that many distributions do not show an obvious orientation but, instead, exhibit an amorphous (roughly circular) pattern and, hence, do not allow speculation concerning historical patterns. As examples, the distribution of the western clade of *M. pallidus* shows an obvious north-west to south-east orientation, yet the distribution of the eastern clade is complex and largely amorphous (Fig. 3). It may be instructive to compare the angular trends derived from haplotype-sharing data with that obtained from all possible pairwise combinations of axial locality data. When this is done for the western clade, there are no significant differences between the orientation trend from the haplotype data and all (120) pairwise locality data (*P*>0.05 for both the Mardia–Watson–Wheeler test and the Watson *U*^2^ test). Non-significant tests here are not surprising, given the general north–south distribution, but this finding does not refute the hypothesis that the orientation patterns reflect historical routes of gene exchange. In contrast, angular distribution tests for the eastern clade show significant differences between the orientation trend from the haplotype-sharing data and data obtained for all possible (66) pairwise combinations of localities (Mardia–Watson–Wheeler *W*=9.905, *P*=0.01; Watson *U*^2^ = 0.241, *P*<0.02). Given its complex shape, inspection of the distribution map of the eastern clade probably would not have predicted the orientation trend from haplotype sharing (Figs 3 & 4b).

### Corroboration of the principal phylogenetic units

The recognition of two basal (eastern and western) lineages within *M. pallidus* based on mtDNA sequence data is corroborated by other studies using different kinds of character set. Hafner’s (1981) study, involving both genetic (isozymic and karyotypic) and phenetic (cranial and external morphometrics and pelage colorimetry) data sets, was first to recognize the eastern and western units within *M. pallidus*. Hafner’s (1981) karyotypic data, although summarizing data from only 10 populations, was perhaps the most definitive of the characters he studied. Hafner (1981) recognized two principal chromosomal forms: a western form, termed the 42-α karyotype (2*n*=42, five pairs of acrocentric autosomes) and an eastern form, the 42-β karyotype (2*n*=42, all bi-armed autosomes). Despite the limited geographical sampling, Hafner (1981) postulated a boundary between these chromosomal forms in south-central Nevada that is near the boundary identified between the two clades of the present study. It should also be noted that Hafner (1981) recognized a third chromosomal form, a 42-γ, described as being similar to the 42-α karyotype and found at the northern edge of the western distribution (localities of Nixon and Wadsworth).

The geographical range of the western clade defined in this study also agrees remarkably well with Hall’s (1941) depiction of the distribution of *M. p. pallidus* based on cranial and external morphology. Indeed, the boundary between Hall’s (1941)*M. p. pallidus* and *M. p. ruficollaris* is nearly coincident with the boundary noted here between the western and eastern clades, respectively; Hall’s representation of the boundary appears to be positioned only about 15–20 km east of the boundary noted here (Fig. 3). Three subspecies of *M. pallidus* from Hall (1941) comprise our eastern clade and, although this does not provide direct support for our eastern clade, Hall’s (1941) recognition of three eastern subspecies is moderately concordant with the genetic subunits that we observe within the eastern clade.

Although Hafner (1981) was able to show multivariate morphological discrimination between most of the eastern and western populations of *M. pallidus* as defined in this study, the kangaroo mice belonging to these clades are nonetheless extremely similar morphologically. It should also be kept in mind that morphological differentiation is slight within the genus and, in fact, the two currently recognized species, *M. pallidus* and *M. megacephalus*, are regarded as classic sibling species (Hafner *et al.*, 1979). Despite the subtle morphological differences between the eastern and western clades of *M. pallidus*, these forms qualify as evolutionarily significant units (for discussion see Moritz, 1994; Blois & Arbogast, 2006). In addition to showing reciprocally monophyletic patterns for mtDNA data, the eastern and western clades show significant divergence in other nuclear markers, especially the karyotypes.

### Cryptic species of kangaroo mice

Average sequence–divergence values for *Cytb* between the eastern and western clades of *M. pallidus* are *c*. 8% (Table 1). As pointed out by Meyer (1994: 278), cytochrome *b* has become an ‘industry standard’ for phylogenetic studies. The level of sequence divergence of the phylogroups of *M. pallidus* exceeds the mean percentage sequence divergence value for sister species reported by Baker & Bradley (2006) and, thus, suggests that the two clades identified here may be genetically isolated species. It should also be noted that our estimation of sequence divergence of *Cytb* is based on only the first portion of the gene. The first section of *Cytb*, known to contain a functioning redox centre in the electron transport chain (Howell, 1989; Irwin *et al.*, 1991), evolves at a slower rate than the second portion of the gene in rodents (Lara *et al.*, 1996; Spotorno *et al.*, 2004) and other mammals (Irwin *et al.*, 1991). As noted by Spotorno *et al.* (2004), reliance on the first portion of *Cytb* leads to an underestimation of genetic divergence. Thus, the *Cytb* percentage sequence–divergence values presented here should be viewed as conservative estimates of genetic divergence between the phylogroups of *M. pallidus*.

It is most likely that the two main phylogroups within the currently recognized species *M. pallidus* represent morphologically cryptic species of kangaroo mice. However, before these clades are recognized taxonomically, research should be conducted at the region of suspected contact (in south-central Nevada; Fig. 3) to determine the nature of the genetic interactions between the forms. Data from this study have already identified a locality, San Antonio (Fig. 3), where both main haplotypic forms are found together, but nuclear markers (e.g. chromosomes and/or allozymes) must be used to determine if the two forms are isolated genetically from each other.

**Figure 5:**
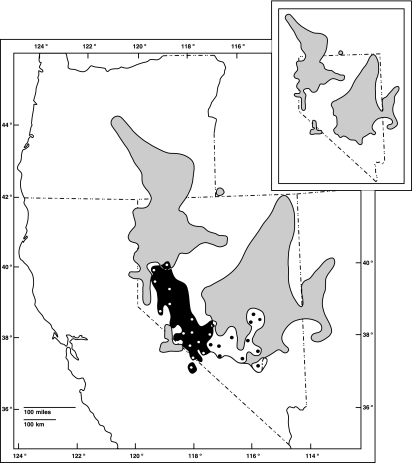
Superimposition of the geographical ranges of *Microdipodops pallidus* Merriam (black and white distributions) and *M. megacephalus* Merriam (grey-shaded distribution) showing the coincidence of the distributional boundary between the eastern and western phylogroups of *M. pallidus* with the distributional border of *M. megacephalus* in south-central Nevada. The inset shows a reduced distribution map of *M. megacephalus* for comparison. The mean elevation of *M. pallidus* localities in the area of overlap of the eastern clade with the distribution of *M. megacephalus* is significantly different (and higher) than the mean elevation of the localities in the western clade (see text for discussion).

From a historical biogeographical perspective, it is difficult to explain what factors may have been responsible for the divergence and geographical placement of the two principal cladistic units of *M. pallidus*. However, we note that there is a chain of north–south trending mountain ranges (the southern end of the Toquima Range, San Antonio Mountains, Lone Mountain, Weepah Hills, Split Mountain, Clayton Ridge and Montezuma Range) that coincides with the boundary of the eastern and western cladistic units. Field reconnaissance and examination of topographic maps indicates that these ranges may represent a physiographic baffle between the clades, limiting dispersal (and presumably gene exchange). Two likely low-elevation routes surmount these ranges, and we have already detected both principal haplotypes near one of these areas (San Antonio locality, Fig. 3). Although we have not assessed possible ecological differences between the habitats on either side of this physiographic baffle, we do note that the mean elevations for localities associated with the eastern (1586 m) and western (1411 m) units are significantly different (*F*=10.525, *P*=0.003) and isophene contours of several climatic characters parallel this north–south physiographic baffle (Houghton *et al.*, 1975). These differences in elevation and climate may signal biotic differences that are important to kangaroo mice. Interestingly, when the distributions of *M. pallidus* and *M. megacephalus* are superimposed (Fig. 5), the border between the eastern and western phylogroups of *M. pallidus* coincides identically with the distributional margin of the range of *M. megacephalus* in south-central Nevada. The coincidence of these boundaries, although indirect evidence, suggests ecological differences in areas to the east and west and, in turn, may indicate differences in the niche of the eastern and western clades of *M. pallidus*.

The divergence of the two main clades of *M. pallidus* may be placed in a temporal context of cladogenic events within the family Heteromyidae (Hafner *et al.*, 2007). Based on fossil calibration of independent molecular sequence data (cytochrome *c* oxidase subunit *I* and 12S and 16S ribosomal RNA genes), Hafner *et al.* (2007) estimated that the divergence of *M. pallidus* and *M. megacephalus* occurred 8.1 Ma. Comparing this time divergence estimate with mtDNA sequence divergence estimates from our study (uncorrected *p* distance for the combined data set; Table 1) yields an estimate of 4.38 Ma as the time of divergence of the eastern and western phylogroups of *M. pallidus*. Hence, divergence between the eastern and western forms occurred at a time (early Pliocene) before the formation of the extensive sandy habitats within the Great Basin by depositional and eolian processes of the Pleistocene and Holocene (Morrison, 1964; Smith, 1982; Mehringer, 1986; Eissmann, 1990). The estimated times of divergence of kangaroo mice and the discovery of fossil kangaroo mice from the late Blancan (about 2.9–1.9 Ma) outside the Great Basin (Remeika *et al.*, 1995; Cassiliano, 1999; Jefferson & Lindsay, 2006) now paint a picture of a relatively ancient heteromyid lineage that did not evolve *in situ* (cf. Hafner, 1978).

## Conclusions

### Applicability of DAPP

The use of angular measurements pertaining to haplotype sharing over geography combined with circular statistical analyses appears to be a promising approach in phylogeographical studies. Orientation data derived from haplotype sharing between pairwise localities provides a means of detecting and quantifying the ‘signatures’ of past events pertaining to movement patterns and gene flow. Each individual distribution map of haplotype sharing between pairwise localities contributes a tiny piece of the geographical history of that matrilineage. However, when angular data are measured for haplotype sharing between all pairwise localities and summarized using the methods of circular statistics, it is possible to quantify patterns of haplotype sharing and subject those patterns to rigorous statistical analysis. As shown in this study, it is possible to test for randomness (uniformity in orientation), calculate a compass trend (a mean vector, μ) that represents a fingerprint of historical routes of gene exchange, and to test for significant differences between two trends. Future workers may want to extend this analysis of orientation data by examining not only shared unique haplotypes, but also orientation data from haplotypes one, two, or three mutational steps removed. Perhaps it is also possible to ascribe an unambiguous direction to the orientation data by including information regarding ancestral haplotypes and outgroup comparisons. The DAPP approach may also be used with other kinds of genetic marker.

### A biogeographical model for sand-obligate organisms

The phylogeographical patterns described here may serve as a model for other kinds of sand-obligate organisms in the Great Basin. Sand-obligate forms, for example, *D. deserti* and the dune-obligate beetle *Eusattus muricatus* LeConte, would be expected to show patterns similar to those described here if they are responding to the same Earth-history events. Key predictions from this study suggest that other sand-obligate forms will show eastern and western phylogroups that diverged about 4 Ma, a contact zone in the south-central region of the Great Basin (vicinity of Tonopah, Nevada), a comb-like pattern of rapid range expansion through the Lahontan Trough in the western unit, and non-random historical routes of gene exchange (specifically, a north-west to south-east orientation in the western clade and a north-east to south-west directional pattern in the eastern clade). Unfortunately, studies addressing the genetic variation of *E. muricatus* (Britten & Rust, 1996; Epps *et al.*, 1998, 2000) did not include thorough sampling across the geographical range of the species and, therefore, comparisons with this study are impossible; however, divergence estimates from allozymic data by Epps *et al.* (1998) suggest divergence times much lower than those estimated here. A comparison between the patterns shown here for *M. pallidus* and *D. deserti* would be particularly interesting as both are sand-obligate heteromyid rodents, but nothing has been published on the phylogeography of *D. deserti*.

## Biosketches

**John C. Hafner** is the Director and Curator of Birds and Mammals in the Moore Laboratory of Zoology and Professor of Biology at Occidental College, Los Angeles. His research focuses on bird and mammal evolutionary biology, particularly the systematics and macroevolution of New World rodents of the superfamily Geomyoidea.

**Nathan S. Upham** is finishing his Master’s degree at Occidental College, where he has been studying the moonlight response of nocturnal desert rodents in the Great Basin. His present research interests are focused on the interaction between past climate fluctuations and speciation processes in mammals.

**Emily Reddington** has interests in bird and mammal biology and is now completing her Master’s thesis at Occidental College on the population genetics and conservation biology of kangaroo mice.

**Candice W. Torres** is currently a doctoral student with research interests in the behaviour, genetics and chemical ecology of ants.

Editor: John Lambshead

## Appendix

Localities and number of specimens examined in this study. Specimens are deposited in either the Moore Laboratory of Zoology (MLZ; Occidental College) or the Museum of Southwestern Biology (MSB; University of New Mexico). Principal localities for *M. pallidus* are shown in bold and are listed alphabetically; principal localities are shown in Fig. 1.

*Microdipodops pallidus* (*n*=98). **ALAMO**: 4.5 miles S, 32.5 miles W Alamo, 4600 feet, Lincoln County, Nevada (*n*=3, MSB 35,536–35,538). **COALDALE**: 1.8 miles S, 5.3 miles E Coaldale, 4797 feet, Esmeralda County, Nevada (*n*=1, MLZ 1817). **CURRANT**: 4.9 miles S, 28.2 miles W Currant, 6000 feet, Nye County, Nevada (*n*=5, MLZ 2000–2004). **DEEP SPRINGS**: 7.2 miles S, 4.0 miles W Deep Springs, 4920 feet, Inyo County, California (*n*=2, MLZ 1767, 1768); 4.6 miles S, 3.9 miles W Deep Springs, 5000 feet, Inyo County, California (*n*=2, MLZ 1769, 1770); 2.4 miles S, 2.3 miles W Deep Springs, 5050 feet, Inyo County, California (*n*=6, MLZ 1771–1776). **DYER**: 7.0 miles N, 0.5 miles W Dyer, 4900 feet, Esmeralda County, (*n*=5, MLZ 1785–1789). **FALLON**: 4.3 miles N Fallon, 3900 feet, Churchill County, Nevada (*n*=1, MLZ 1947). **GOLDFIELD**: 12.0 miles N, 2.5 miles W Goldfield, 4860 feet, Esmeralda County, Nevada (*n*=2, MLZ 1743, 1746). **SE GOLDFIELD**: 4.6 miles S, 19.8 miles E Goldfield, 4950 feet, Nye County, Nevada (*n*=2, MLZ 2051, 2052). **GOLD REED**: 3.0 miles S, 4.3 miles E Gold Reed, 5330 feet, Nye, County, Nevada (*n*=2, MLZ 1958, 1959). **W HIKO**: 6 miles N, 31 miles W Hiko, 4800 feet, Lincoln County, Nevada (*n*=4, MLZ 1811–1814). **LOCKES**: 9.6 miles S, 3.8 miles W Lockes, 4800 feet, Nye County, Nevada (*n*=4, MLZ 2017–2020). **LOVELOCK**: 2.5 miles N, 22.5 miles W Lovelock, 3950 feet, Pershing County, Nevada (*n*=1, MLZ 1967). **LUNING**: 9.8 miles N, 10.8 miles E Luning, 5350 feet, Mineral County, Nevada (*n*=5, MLZ 1805–1809); 12.7 miles N, 9.2 miles E Luning, 5050 feet, Mineral County, Nevada (*n*=1, MLZ 1810). **MARIETTA**: 0.4 miles S, 0.5 miles E Marietta, 4950 feet, Mineral County, Nevada (*n*=3, MLZ 1777–1779). **MINA**: 8.9 miles S, 1.2 miles E Mina, 4400 feet, Mineral County, Nevada (*n*=5, MLZ 1780–1784). **NEW REVEILLE**: 0.9 miles N, 10.3 miles E New Reveille, 4900 feet, Nye County, Nevada (*n*=2, MLZ 1940–1941). **NIXON**: 6.4 miles N, 1.0 miles W Nixon, 4200 feet, Washoe County, Nevada (*n*=1, MLZ 1794). **OASIS**: 0.2 miles S, 1.5 miles E Oasis, 5050 feet, Mono County, California (*n*=2, MLZ 1790, 1791); 1.0 miles S, 4.0 miles E Oasis, 5100 feet, Mono County, California, (*n*=2, MLZ 1792, 1793). **SAN ANTONIO**: 0.5 miles S San Antonio, 5400 feet, Nye County, Nevada (*n*=3, MLZ 1796–1798). **SCHURZ**: 7.3 miles N, 2.6 miles W Schurz, 4287 feet, Mineral County, Nevada (*n*=3, MLZ 1818–1820). **SILVER PEAK**: 5.1 S, 1.1 miles E Silver Peak, 4300 feet, Esmeralda County, Nevada (*n*=2, MLZ 1945, 1946). **E TONOPAH**: 0.5 miles N, 32.0 miles E Tonopah, 5600 feet, Nye County, Nevada (*n*=4, MLZ 1801–1804). **NW TONOPAH**: 9.2 miles N, 8.1 miles W Tonopah, 4850 feet, Nye County, Nevada (*n*=1, MLZ 1973). **SE TONOPAH**: 11.0 miles S, 10.0 miles E Tonopah, 5200 feet, Nye County, Nevada (*n*=5, MLZ 1821–1825); 10.6 miles S, 10.0 miles E Tonopah, 5200 feet, Nye, County, Nevada (*n*=5, MLZ 1826–1830). **WADSWORTH**: 1.0 miles N, 1.0 miles W Wadsworth, 4200 feet, Washoe County, Nevada (*n*=1, MLZ 1795). **NE WARM SPRINGS**: 19.2 miles N, 13.4 miles E Warm Springs, 6000 feet, Nye County, Nevada (*n*=5, MLZ 1906, 1952–1955). **YERINGTON**: 11.7 miles S, 3.5 miles E Yerington, 4690 feet, Lyon County, Nevada (*n*=3, MLZ 1832–1834); 11.1 miles S, 2.8 miles E Yerington, 4640 feet, Lyon County, Nevada (*n*=5, MLZ 1835–1839).

*Microdipodops megacephalus*. 5.3 miles S, 1.6 miles E Geyser, 5900 feet, Lincoln County, Nevada (*n*=1, MLZ 1974); 3.7 miles N, 3.2 miles E San Antonio, 5600 feet, Nye County, Nevada (*n*=1, MLZ 1762); 5.5 miles S, 9.2 miles W Winnemucca, 4300 feet, Humboldt County, Nevada (*n*=1, MSB 35535).

*Dipodomys deserti*. 10.7 miles S, 25.0 miles W Gerlach, 3950 feet, Washoe County, Nevada (*n*=1, MLZ 2065).

*Dipodomys microps*. 6 miles N, 0.5 miles W Bishop, 4200 feet, Inyo County, California (*n*=1, MLZ 1765).
